# Biopolymer Non-Parametric Analysis: A Degradation Study under Accelerated Destructive Tests

**DOI:** 10.3390/polym15030620

**Published:** 2023-01-25

**Authors:** Elias H. Arias-Nava, Delia J. Valles-Rosales, B. Patrick Sullivan

**Affiliations:** 1 Department of Industrial Engineering & Operations, Instituto Tecnologico Autonomo de Mexico, Mexico City 01080, Mexico; 2 Department of Industrial Management & Technology, Texas A&M University-Kingsville, Kingsville, TX 78363, USA; 3 Department of Design Production, University of Twente, 7522 Enschede, The Netherlands

**Keywords:** non-parametric analysis, mechanical testing, statistical analysis, accelerated lifetime testing, lifetime analysis, PLA

## Abstract

The degradation of biopolymers such as polylactic acid (PLA) has been studied for several years; however, the results regarding the mechanism of degradation are not completely understood yet. PLA is easily processed by traditional techniques including injection molding, blow molding, extrusion, and thermoforming; in this research, the extrusion and injection molding processes were used to produce PLA samples for accelerated destructive testing. The methodology employed consisted of carrying out material testing under the guidelines of several ASTM standards; this research hypothesized that the effects of UV light, humidity, and temperature exposure have a statistical difference in the PLA degradation rate. The multivariate analysis of non-parametric data is presented as an alternative to multivariate analysis, in which the data do not satisfy the essential assumptions of a regular MANOVA, such as multivariate normality. A package in the R software that allows the user to perform a non-parametric multivariate analysis when necessary was used. This paper presents a study to determine if there is a significant difference in the degradation rate after 2000 h of accelerated degradation of a biopolymer using the multivariate and non-parametric analyses of variance. The combination of the statistical techniques, multivariate analysis of variance and repeated measures, provided information for a better understanding of the degradation path of the biopolymer.

## 1. Introduction

Polylactic acid (PLA) is a biodegradable polymer made from renewable resources such as corn and potato starch, as well as sugars generated from beets, cane, and other agricultural goods. PLA may be utilized in a wide range of products due to its ability to be easily processed using traditional methods such as injection molding, blow molding, extrusion, and thermoforming, as well as its high strength and thermo-plasticity. Gupta and Kumar [1] found that, depending on the use for various products, PLA is well suited and commonly used due to its low molecular weight and reduced degradation time. PLA has a reasonable shelf-life for most single-use packaging applications, meaning that the products are used once, or for a short period of time, before being thrown away. There are different applications for PLA, as rigid plastics, biaxially oriented films, plastic bottles, meat trays, opaque dairy (yogurt) containers, consumer displays, electronics packaging, envelop and display carton windows, bottles for short-shelf-life milk, and bottles used for edible oils [2]. PLA and its copolymers have attracted significant attention in environmental, automotive, biomedical, and pharmaceutical applications as alternatives to petroleum-based polymers due to their mechanical and physical properties and, in particular, their short degradation time [3]. The use of PLA could be a technical and economic solution to the problem of the eventual disposal of the very large amount of plastic packaging used in the USA [4].

PLA degradation has been studied for several years; however, the results regarding the mechanism of degradation are not completely understood yet [5]. The investigation of the degradation rates of biopolymers would allow industries and researchers to predict the life-span of these types of materials and learn how to further improve a product’s usable life. In terms of production costs, PLA presents advantages such as energy savings between 25 and 55 percent compared to petroleum-based polymers; up to this point, the challenge has been reducing PLA’s manufacturing cost. Since the use of PLA products continues to grow, it is useful to understand the degradation rates by studying the accelerated failure process. This process is called accelerated life testing, and it requires a model representation [3,6]. A powerful knowledge/understanding tool based on degradation models could, then, lead to the predictability of the usable life of materials and products. In general, most polymers experience degradation due to a combination of two or more factors such as heat, light, oxygen, and/or water. The degradation level of a polymeric material depends on its ability to absorb UV light (due to the presence of catalyst residues such as hydro peroxide and carbonyl groups) and/or water. The exposure of polymers to UV light irradiation leads to chain scission, which causes mechanical deterioration and the breaking of the material into small pieces; this consequently allows oxygen and microorganisms to degrade the polymer at an accelerated rate [7].

Test responses could be similar among different models of acceleration, depending on the specific test used during the experimentation. The main difference is that the different statistical analyses of the results may lead to a different statistical model [8]. Several studies related to the degradation model have been performed and analyzed, some of them with characteristics similar to the type of degradation. A previous study of degradation analysis listed eight different approaches, which were used as the baseline in the methodology of this research, including the accelerated destructive degradation (ADDT) approach [9].

## 2. State-of-the-Art

### 2.1. PLA Degradation Testing

PLA is water-insoluble when it has a sufficiently high molecular weight. When water penetrates the polymer matrix and hydrolysis on the ester groups takes place, long polymer chains are converted into short ones; subsequently, oligomers and monomers are created as a result of the water solubility of the polymer. The degradation of PLA is affected by several factors: material, hydrolysis media, as well as by the factor’s coefficient such as molar mass, degree of swelling of the matrix, rigidity, chemical structure, molecular weight, chain mobility, and crystallinity [10]. Some studies presented results indicating apparent visual signs of degradation; however, no significant weight loss was presented within 39 weeks in compost. This situation suggested that degradation happened from the interior of the samples. The molecular weight decreased to around 36–44% after the melting process. The average molecular weight of PLA decreased from 149,593 to 113,096 g/mol [11]. In a previous study, experimentation with different commercial polymers was performed, and it was concluded that the degradation occurs due to chain scission of these polymers when they are exposed to UV. It is well known in polymer science that the exposure of polymers to UV light irradiation leads to main chain scission, causing mechanical deterioration, breaking into small pieces, and consequently, oxygen and microorganism access, making the polymer biodegradable. This information is extremely relevant when UV light experimentation is being considered [12].

The understanding of the degradation, as well as the impact of the use of thepolymers is a relevant topic in the manufacturing industry; the environment where polymers are used must not be negatively impacted. Both terrestrial and marine ecosystems need to be preserved in their natural equilibrium. In fact, two strategies are being studied for the production of biodegradable polymers with short lives, such as for food packaging, agricultural mulches, medical devices, etc., as well as innovative technologies. It might be challenging to strike the ideal balance between long-lasting and biodegradable polymers. The new materials are expected to be durable while in use, yet biodegradable when their useful lives are through. Co-extrusion of natural and man-made polymers is one potential technique [13].

It is important to point out that previous studies have created a path toward degradation analysis of biopolymers, such as PLA, which includes tensile and flexural testing as the main indicators of the degradation of the materials. Mechanical properties are very important in the manufacturing industry because they are, sometimes, the key to product durability. Understanding and improving the mechanical properties of biopolymers over time is one of the researchers’ shared goals in terms of polymer characteristics and characterization. Mechanical testing, on the other hand, is simply one of the features used to analyze the material’s capabilities in current biomaterials. Based on this, the current study aimed to improve the capability of biopolymer analysis by adding a variety of tests to the analysis, allowing for a better understanding and more accurate degradation predictions [14].

### 2.2. Accelerated Testing

Degradation has many different definitions depending on the experiment or situation that is being evaluated. In the field of reliability, this means making “time” go faster so that inference about the material or product can be made rapidly. There are several methods of acceleration depending on the specimen that is being analyzed: One is to increase the use rate of the product; this method applies when the product/material is not in continuous use. Other methods are to increase the intensity of the radiation exposure, increase the level of stress (voltage, pressure, etc.), or increase the aging rate of the product; these methods increase the level of the experimental variables such as the temperature, humidity, and UV light, and these types of acceleration lead to a faster degradation rate of the chemical and physical processes of failure. Furthermore, a combination of these methods is used depending on the study [8].

Escobar described that, depending on the type of accelerated testing used in the experimentation, there may be a different type of response, and some commonly used testing types are described as follows:

1. *Accelerated binary tests (ABTs)*: The response is binary; there is only one thing to evaluate: whether the product has failed or has not failed.
2. *Accelerated life tests (ALTs)*: The response is directly related to the lifetime of the product, based on inspections at intervals, and the failure may or may not happen in one interval.
3. *Accelerated repeated measures degradation tests (ARMDTs)*: In this type of test, degradation is measured on a sample of units at different points in time; one unit may provide many degradation measures (usually depending on whether the test is destructive or not); in this case, the degradation response is open to the researcher; it may be physical or chemical changes, color changes, mass loss, or any particular characteristic of the product itself.
4. *Accelerated destructive degradation tests (ADDTs)*: An ADDT is similar and can be used as a complement to an ARMDT, except that the measurements of the response are destructive; some examples of this kind of test are tensile and flexural tests, which completely break the specimens in order to estimate the yield points [8].

## 3. Materials and Methods

The goal of this research was to present a multivariate non-parametric analysis of the lifetime of PLA. The experiment’s general hypothesis (Hypothesis 1) was that the accelerating factors of UV light, humidity, and temperature have a statistical difference in the degradation rate of PLA after an accelerated weather exposure of 2000 h. In Hypothesis 2, the experiment evaluated four response factors individually to determine if they were affected by the exposure of 2000 h. Finally, the third hypothesis stated that the acceleration time has a significant effect on the PLA samples, after 250, 500, 1000, and 2000 h of accelerated condition exposure.

The research methodology utilized a series of experiments to test the proposed hypotheses. The experiments were carried out under the guidelines of the American Society for Testing and Materials (ASTM) standards: ASTM D618-05 Standard Practice for Conditioning Plastics for Testing is a test standard that considers that the the physical and electrical properties of plastics are influenced by temperature and relative humidity in a manner that materially affects the test results. In order for reliable comparisons to be made of different materials and between different laboratories, it is necessary to standardize the humidity conditions, as well as the temperature to which the specimens of these materials are subjected prior to and during the testing. ASTM D638-10 Standard Test Method for Tensile Properties of Plastics is a test method covering the determination of the tensile properties of unreinforced and reinforced plastics in the form of standard dumbbell-shaped test specimens when tested under defined conditions of pretreatment, temperature, humidity, and testing machine speed. The test data obtained by this test method are relevant and appropriate for use in engineering design. This test method is designed to produce tensile property data for the control and specification of plastic materials. ASTM D790-03 Standard Test Methods for Flexural Properties of Unreinforced and Reinforced Plastics and Electrical Insulating Materials is a test method that covers the determination of the flexural properties of unreinforced and reinforced plastics, including high-modulus composites and electrically insulating materials in the form of rectangular bars molded directly or cut from sheets, plates, or molded shapes. ASTM G151-00 Exposing Nonmetallic Materials in Accelerated Test Devices that Use Laboratory Light Sources, is a practice that provides the general procedures to be used when exposing nonmetallic materials in accelerated test devices that use laboratory light sources. Detailed information regarding the procedures to be used for specific devices is found in the standards, describing the particular device being used. ASTM G155-04a Standard Practice for Operating Xenon Arc Light Apparatus for Exposure of Non-Metallic Materials is a practice that covers the basic principles and operating procedures for using xenon arc light and a water apparatus intended to reproduce the weathering effects that occur when materials are exposed to sunlight (either direct or through window glass) and moisture as rain or dew in actual use. This practice is limited to the procedures for obtaining, measuring, and controlling the conditions of exposure. Test specimens are exposed to filtered xenon arc light under controlled environmental conditions. Different types of xenon arc light sources and different filter combinations are described. Finally, ASTM D2565-08 Standard Practice for Xenon-Arc Exposure of Plastics Intended for Outdoor Applications is a practice that covers the specific procedures and test conditions that are applicable to xenon arc exposure of plastics conducted in accordance with Practices G 151 and G 155. This practice also covers the preparation of the test specimens, the test conditions best suited for plastics, and the evaluation of the test results. Significance and use: the ability of plastic material to resist the deterioration of its electrical, mechanical, and optical properties caused by exposure to light, heat, and water can be very significant for many applications. This practice is intended to induce property changes associated with end-use conditions, including the effects of daylight, moisture, and heat.

The setup of the parameters and the fabrication process: In order to create samples for flexural and tensile testing, a number of trials were run to determine the initial parameters of the extrusion and injection molding processes. The following conditions were specified for the last trial for which the samples were created: The following process conditions were used: the DMS extruder melt temperature for PLA was set at 200 °C; the motor speed was set at 50 Rpm; the max force was 8000 N; the max acceleration speed was 1000 Rpm. For the injection molding: the mold temperature was set at 45 °C; the molding temperature was set at 175 °C. The process was as follows: Step 1, 12 bar for 13 s; Step 2, 12 bar for 12 s. Ultimately, a total of 180 good PLA flexural samples were fabricated using these parameters. Another set of trials was performed to fabricate the samples for tensile testing. Adjustments were needed of the injection molding machine for the tensile samples as follows: injection molding for tensile testing: the melting temperature was set at 180 °C; the mold was set at 55; Process Step 1, 12 bar for 10 s; Step 2 12 bar for 6 s. Finally, a total of 90 tensile samples were fabricated using these parameters.

### Experimentation

**Figure A1:**
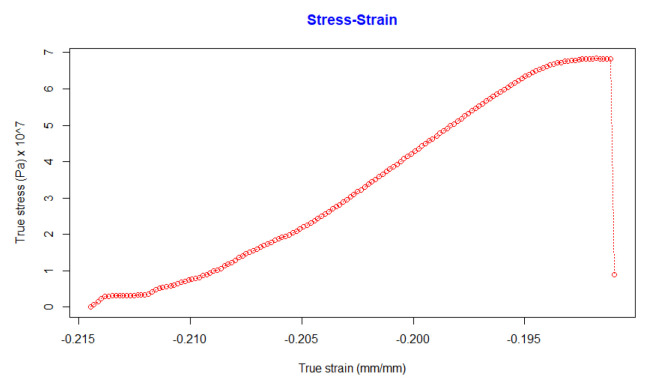
Strain–stress for tensile testing.

The experiment included 10 replications on each test/code; ASTM standard recommends at least 6 specimens to validate the experiment, and it was decided to include 10 to minimize the variability. The sample fabrication included a total of 70 color samples, 70 flexural samples, and 70 tensile samples. Another 30 extra samples (10 for each group of tests) were fabricated to be tested as control samples. The times selected were based on the methodology and previous experiments in the area of degradation [3]. The accelerated destructive degradation test (ADDT) approach was the approach used in this paper, and it was proposed by Meeker et al. [15]. In an ADDT type of test, the measurements of the response are destructive; as examples of this kind of test, we can mention that the tensile and flexural tests completely break the specimens in order to estimate the yield points. Temperature (which is specified by ASTM) was set at room temperature for the tensile and flexural testing, between 20 °C and 30 °C. Additionally in Appendix C, a strain–stress graph is added for the purposes of the illustration of the experimentation (Figure A1).

The necessity of extrapolating data is a well-known feature of accelerated testing, and the experiment is typically carried out under accelerated conditions; however, the results are intended to provide information for real or natural situations [16]. It was expected that PLA would have a rapid visual degradation (250 h), and then, the degradation rate would slow down, which is the reason for the following times being in 500 h’ intervals (500, 1000, 1500, and 2000). The fabrication process was performed using an extrusion and injection molding machine; the extrusion machine had 15cc twin co-rotating screws by the Xplore model DSM 15 cc capacity. The machine’s control was based on two sections: the front and rear section with three heating areas: up, middle, and low-out section. The injection molding machine was an Xplore DSM 12 cc heating chamber, model Micro 12 cc IMM. Subsequently, the PLA samples were exposed to accelerated degradation using the weatherometer ATLAS Ci5000 Xenon Weather-Ometer.

**Figure 1:**
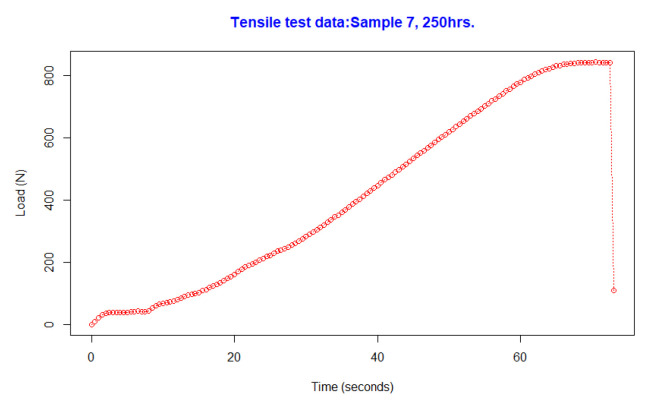
Tensile test.

Tensile testing was performed at different points of time, and the machine used was the INSTRON 5882 Floor Model Testing Systems (100,000 N (22,500 lb.)). This equipment performs tensile and compression testing. Data points were collected in Newtons (load). The results showed that, for instance, in Sample 7, the breaking point was reached at 70.5 s and 842.5 Newtons, and the test is graphically represented in Figure 1. The graph presents the load in Newtons (N) required to break the tensile samples, and the x-axis is the time the machine took to break the part in seconds (s).

Batch 1 was a control group averaged at a UTS of approximately 85 MPa. The UTS of this material was slowly decreased, assumed to be affected by the exposure to the accelerating factors of UV, humidity, and high temperatures. Flexural testing was performed by using a three-point test. The machine used was the INSTRON 5882 Floor Model Testing Systems. The maximum stress and strain are calculated on the incremental load applied, and this was used in the experimentation of this research. In the flexural testing, the bar samples (fabricated under ASTM D790) were positioned as a flat cross-section on two parallel supporting pins. The load was then applied through a loading point in the center of the cross-sectional area of each sample. In this study, what was important was the force needed to break the sample (since degradation is being evaluated); however, we included a strain–stress graph in Appendix C for the tensile testing.

**Figure 2:**
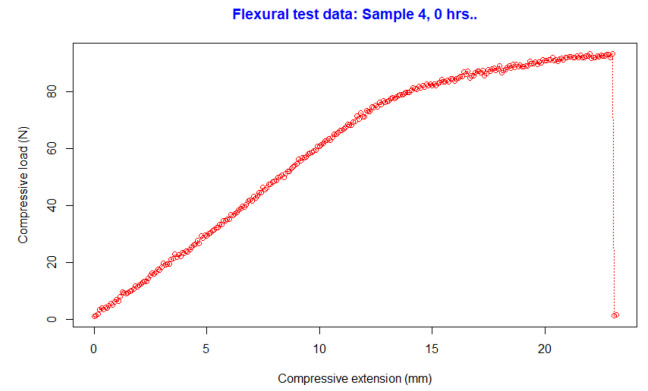
Flexural test.

The flexural samples were tested until they reached the breaking point. The maximum force at the breaking point was evaluated, and the flexural strength was calculated. Figure 2 presents the force applied and the breaking point in N (for Sample 4 at 0 h). The data were used to calculate the flexural strength when the material broke. In this experimentation, all samples broke during the tests; it is important to mention that some polymeric materials with high elasticity usually just bend at their maximum force and do not break, and this is relevant for researchers that are looking to experiment with different ranges of brittleness of the material.

**Figure 3:**
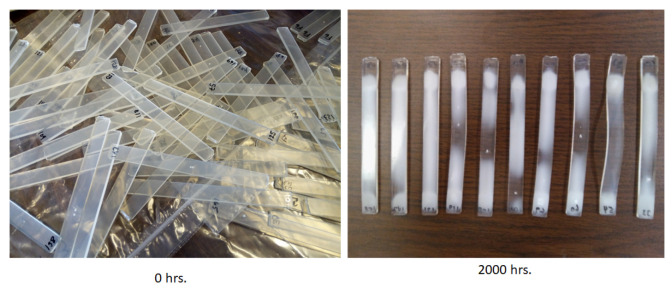
Degradation.

For the non-parametric analysis, the findings of the flexural strength (measured in megapascals) for each sample tested under these conditions were determined. The L*a*b* colorimetry model devised by the Commission International d’Eclairage (CIE) was used for color testing. In a technical report publication (15.2), they established color testing as a standard (1986). Color denotes lightness and is described by two axes: The L* (lightness) axis, which goes from 100 to zero, with 100 denoting perfect reflecting diffusion and zero denoting dark color. The a* and b* axes are the two chromatic components (ranging from −120 to 120), and the a* component goes from green (−120) to red (120), while the b* component goes from blue (−120) to yellow (120). The results for the 60 samples were used for the analysis. Weight loss (also known as mass loss) in a material is closely related to the strength and flexibility of materials. Samples were weighed before conducting the destructive flexural test. The initial weight of the control samples averaged 7.076 gr. These data were used to analyze the behavior of the material during the 2000 h of accelerated weathering exposure. Physical evidence of the changes in the material are presented in Figure 3 for illustration purposes.

## 4. Statistical Analysis

Multivariate analysis of variance (MANOVA) can be expressed as a set of tools that provide simultaneous analysis of multiple dependent and independent variables; more in general, a design is considered multivariate if it involves two or more dependent measures. A multivariate test is designed to account for multiple responses and combines them into a single analysis. Suppose that you have six dependent variables, and you want to run an ANOVA, then six different tests would be necessary. MANOVA is able to do this in one test and infer more information among the variables. The dependent variables that are included in the analysis are often called a “composite variable” or a “synthetic variable”, and in such a way, a new variable is created; then, the test is performed on this composite variable, and inferences are made based on this new variable [17]. Rencher and Christensen explained that multivariate analysis consists of a collection of methods that can be used when several measurements are made on each individual or object (research units, experimental units, or sampling units) in one or more samples. Historically, multivariate analysis has had applications in behavioral and biological sciences; currently, this technique is used in multiple science fields including chemistry, education, physics, geology, medicine, law business, mining, engineering, and many more fields [18].

Repeated measures or repeated measurements analysis designs are defined by measures taken on each participant (sample unit) under each of several conditions; the conditions could refer to a situation or very commonly to different times. This strategy is widely used by researchers, mainly because it offers a more powerful analysis than between-subject designs, offering a greater likelihood of rejecting a false null hypothesis; the statistical reason for this situation is that the samples are their own control, and variation due to individual difference is one of the components of the error in the variance “noise”. In experimentation and statistical analysis, no matter the design or analysis that is being used, the researcher is always looking for ways to reduce the error in the variance [18]. In many cases, because the multivariate analysis allows the researcher to carry out a specific test of the experimental hypothesis, it might actually be more powerful than the traditional statistical univariate test analysis [19].

In experimentation, it is common to encounter a problem that involves several responses of the same experimental unit (ex: person, animal, machine, final product, materials). These response variables usually represent different qualitative characteristics; mass loss, length, width, color change, etc. Variation among these variables and the significance of the experimental outcome can be measured by the use of multivariate methods, such as multivariate analysis of variance. Along with this, these variables might be coming from a one-time measure, which would be a simple MANOVA, or the measurements may come from performing a repeated measures response to the levels of an experimental factor of interest, such as time, treatment, or dose; in such a case, repeated measures would be necessary [20]. MANOVA and repeated measures comprise the base methodology for the experimentation in this research.

### Non-Parametric Analysis

The multivariate analysis of non-parametric data is presented as an alternative testing to multivariate analysis when the data do not satisfy the essential assumptions of regular MANOVA, such as multivariate normality; sometimes, the restrictions of the classical parametric MANOVA are very hard to verify, making the analysis useless in such cases. Another restriction of MANOVA is that, even when the normality assumptions are met, the analysis is hard to use because only a global statement about the significance is calculated, and there is a lack of information related to the sub-groups of the response that affect the global significance. Burchett et al. [16] developed a package in the R software that allows the user to perform a non-parametric multivariate analysis, when necessary. They intended to solve two critical issues of the regular MANOVA: (a) providing a fully non-parametric approach, and even more important for this research, (b) providing a procedure to identify the significance response at variable levels. The non-parametric package is *“npmv”*. The*“npmv”* package states that the multivariate observation vectors *Xij = (Xij(1) , …, Xij(p))*T are independent, and within the same factor level i, and they follow the same p-variate distribution: *Xij Fi.* It is assumed implicitly that the same p response variables are observed in the same levels, and that these p variables may be dependent.

Burchett et al. [16] described that the hypothesis testing can be statistically formulated as follows: Ho: F1 = . . . = Fa. If the global hypothesis is rejected, further analysis is desired. The first thing you want to know in the follow-up analysis is which variables or treatments, or even groups, contribute to the significance of rejecting the null hypothesis, and the npmv package performs this type of analysis. There are several ways to test the overall null hypothesis of the multivariate distribution. Using the “npmv” package, four different tests were performed; they were based on the theoretical/mathematical array in several previous studies [21,22]. The tests performed with this package were an approximation of the multivariate analysis of variance. The approximations used for hypothesis testing were the ANOVA type; Wilk’s Lambda type; Lawley–Hotelling type (McKeon’s F approximation); and Bartlett–Nanda–Pillai type (Muller’s F approximation).

Grimm and Yarnold [17] pointed out that the approach of using a MANOVA in repeated measures creates no strategic complications in comparison to using repeated measures ANOVA. However, to fit repeated measures data into a MANOVA framework, it is necessary to create a set of dependent variables. It is helpful to understand the main differences between ANOVA and MANOVA in repeated measures; it is the handling of the within-subject effect. The MANOVA approach for repeated measures can offer advantages over the traditional mixed-model analysis. In many cases, because the multivariate analysis allows the researcher to carry out specific tests of the experimental hypothesis, it might actually be more powerful than the traditional statistical univariate test analysis.

## 5. Results and Discussion

The database consisted of the independent variable (time) and four response variables: flexural strength, ultimate tensile strength, weight (W), and color (L). A sample of the database is presented in Table 1. There were three initial tests that were conducted for this multivariate database: (1) based on the hypothesis that at least one group (time) is statistically significantly different from the other group (see Section 3); (2) individual responses analysis; and (3) looking for multivariate significance. The analysis was performed using the R software.

As stated in the goals presented for this paper, Hypothesis # 1 was set to determine if there was a significant difference in the multivariate analysis of variance. Here, four different tests were presented: Wilks, Pillai, Hotelling–Lawley, and Roy tests. All the test results had a *p*-value less than 0.05, therefore rejecting the null hypothesis Ho. This indicates that the multivariate analysis of variance is statistically significant, in other words that the accelerating factors of UV light, humidity, and temperature had a statistical difference in the degradation rate of PLA after an accelerated weather exposure of 2000 h. The R software results are presented in Table 2.

The individual tests for the four responses were conducted in order to identify what variables were significant in the statistical difference of the multivariate analysis. The results indicated that three of them were significant. The tensile test, the flexural test, and the color test had *p*-values of 1.41 × 10${}^{-6}$, 5.18 × 10${}^{-9}$, and 1.52 × 10${}^{-6}$ , respectively. Weight (mass) loss resulted in being not statistically significant different during the accelerated exposure with a *p*-value of 0.4204. Individual results from the R software are presented in Table 3. For these particular results, the *p*-value for UV light degradation rate was used to test what factor was affected by the accelerated weather exposure of 2000 h; individually, only weight loss was not affected.

A follow-up analysis was conducted to determine if the data followed a multivariate normal distribution. For this, a skewness and kurtosis test was conducted, and the null hypothesis for this test was that “the sample data are not significantly different than a normal population”. In this case, probabilities greater than 0.05 indicated that the data were coming from a normally distributed population. Probabilities less than 0.05 indicated that the data were not normally distributed. The *p*-values for both tests were less than 0.05, indicating that the data were not normally distributed. Follow-up tests for individual normality were conducted (as suggested by Shapiro–Wilk). The results indicated that a *p*-values less than 0.05 suggested that none of the responses were following a normal distribution. The results are presented in Table 4.

With these results, it can be concluded that MANOVA may not be the best way to analyze the degradation test results of this experimentation. One of the restrictions of using a multivariate analysis of variance is that, in order to provide reliable results, the data should come from a normally distributed population.

Based on these results, a different approach must be considered to analyze the results for multivariate analysis. The non-parametric inference tests for multivariate data are presented as an alternative to a regular MANOVA, and as mentioned before in this document, these tests do not require that the data come from a normally distributed population. The functions nonpartest and ssnonpartest in the R software were used to calculate the non-parametric test statistics; nonpartest was used to conduct the global hypothesis test for multiple responses, as well as to provide certain information for every response variable [22]. In the first step, the software assumes that the data have no missing values (meaning all rows and columns are complete in the database). No missing values comprise one of the restrictions, and all groups should be measured at the same points in time, as suggested by Burchett et al. [16]. In this case, all four tests were selected for calculation: ANOVA type approximation, Lawley–Hotelling test approximation, Bartlett–Nanda–Pillai test (Muller’s approximation), and Wilks Lambda, as shown in Table 5.

The results indicated that the effect of the independent time variable or treatment effects was highly significant. At this point, the multivariate test was significant among all three remaining variables. Next, it is essential to know which variables were in fact contributing to this global difference. ssnonpartest provides a more detailed comparison of the different variables involved in the multivariate test. The software package uses an algorithm that determines which of the variables contribute to the significant differences [16]; this follow-up test was intended to indicate if the response variables were statistically significantly different at the various points of the independent variable time at 0, 500, 1000, 1500, and 2000 h.

The R software computes all comparisons through the database, i.e., 0 vs. 2000, 0 vs. 1500 vs. 2000, etc. The R code used is presented in Appendix A. The Wilks’ Lambda type statistic was used in the following test. If the global hypothesis was significant, then the subsets of the algorithm continue. The results are shown in Appendix B, where all appropriate subsets using the factor levels were checked using a closed multiple-testing procedure, which controls the maximum overall Type I error rate at alpha = 0.05. All comparisons among the different points of time rejected the null hypothesis, concluding a statistically significant difference. The third hypothesis was rejected, and it was concluded that acceleration time had a significant effect on the PLA samples, after 250, 500, 1000, and 2000 h of accelerated exposure.

## 6. Closing Remarks and Conclusions

There were three hypotheses stated in this research paper, and each hypothesis served to test a part of the experimentation, as well as the changes (degradation) of the material through the accelerated weathering conditions. In the first hypothesis, the goal was to identify if the selected response variables were significantly affected by the designated accelerated degradation variables: UV light, humidity, and temperature. The hypothesis testing was performed by using the multivariate non-parametric test. Table 1 presented a multivariate test with four multivariate tests: ANOVA type test, McKeon approximation for the Hotelling test, Muller approximation for the Bartlett–Nanda–Pillai test, and Wilks Lambda test. All four tests provided a *p*-value less than 0.001; with the small *p*-value, the null hypothesis is rejected. It was concluded that there was a statistically significant effect of UV light, humidity, and temperature on the degradation rate of PLA after 2000 h of accelerated weather exposure [14].

The second hypothesis targeted the effect on the response variables, analyzed individually, concluding that, out the four responses, tensile, flexural, lightness and weight loss, only three of these had a statistically significant effect. A note here is related to the methods used, different from what normally is used for testing the mechanical properties of biopolymers: by the integration of various factors affecting the material and measuring (multivariate) as would be the reality of a product made of a biopolymer or any material, the purpose is not to focus on the effect on one particular property, but to widen the range. This research paper is part of a more complete research work in which the degradation model were proposed in another paper (by the same authors) [9].

The third hypothesis was related to the different times selected for the experiment. It is necessary to know the point of time at which the acceleration time has an effect on the PLA samples. The test included the degradation after 250, 500, 1000, 1500, and 2000 h of accelerated conditions exposure. The testing for this hypothesis was, in fact, a follow-up test of Hypothesis 1. After testing the overall significance and by using the function ssnonpartest, a series of tests was conducted. The test looked for statistical differences among the six different times. The test was conducted, and the null hypothesis was rejected. It can be concluded that there was a statistically significant effect of the acceleration time on the PLA samples after 0, 250, 500, 1000, and 2000 h of exposure.

The findings show that PLA’s characteristics caused its flexural strength to degrade more quickly than its tensile strength. This biopolymer has a tendency to be fragile, according to the literature. The study’s findings quantified and modeled this particular trait. The action of UV radiation on the material can be used to explain why, in terms of physical qualities, the material loses its lightness before it loses weight.

The duration of the research’s experiments was restricted by the equipment resources’ availability. Using the same setup parameters with a longer exposure duration as the next stage in this research could produce findings that are more accurate. The experiment, for instance, may run 3000 h and involve at least ten separate points where the material degradation would be assessed. The reliability and longevity of one biopolymer were thoroughly investigated in this work through experimental methods. It is recommended to generalize the research’s technique to other biopolymers with comparable mechanical and chemical properties.

## Figures and Tables

**Table 1:** Accelerated degradation test database.

Batch	Sample	Time (h)	Tensile (Mpa)	Flexural (Mpa)	W (g)	L*
1	1	0	84.73	131.90	7.09	80.06
1	2	0	85.50	127.78	7.05	80.64
1	3	0	86.24	121.32	7.05	80.8
1	4	0	84.75	121.90	7.07	79.95
1	5	0	86.60	125.80	7.09	80.23
…	…	…	…	…	…	…
6	58	2000	67.1835	20.8572	7.06	56.17
6	59	2000	82.6966	12.4594	7.03	53.78
6	60	2000	64.329	33.2392	5.88	68.01

**Table 2:** MANOVA: R *p*-value results.

Multivariate Analysis of Variance Tests						
summary(fit, test=“Wilks”)						
	Df	Wilks	approx F	Df	den	Pr(>F)
group	5	0.15739	6.3473	20	170.1	1.51 × 10${}^{-12}$
Residuals	54					
summary(fit, test=“Pillai”)						
	Df	Pillai	approx F	Df	den	Pr(>F)
group	5	1.1179	4.1888	20	216	4.58 × 10${}^{-8}$
summary(fit, test=“Hotelling–Lawley”)						
	Df	Hotelling–Lawley	approx F	Df	den	Pr(>F)
group	5	3.7475	9.275	20	198	2.2 × 10${}^{-16}$
Residuals	54					
summary(fit, test=“Roy”)						
	Df	Roy	approx F	Df	den	Pr(>F)
group	5	3.3194	35.85	5	54	5.39 × 10${}^{-16}$
Residuals	54					

**Table 3:** Individual variables’ analysis of variance.

Response Variables					
Tensile					
	Df	Sum	F	value	Pr (>F)
group	5	4268.7	853.75	9.5655	1.41 × 10${}^{-6}$
Residuals	54	4819.6	89.25		
Flexural					
	Df	Sum	F	value	Pr(>F)
group	5	84625	16924.9	14.525	5.18 × 10${}^{-9}$
Residuals	54	62922	1165.2		
Weight					
	Df	Sum	F	value	Pr(>F)
group	5	0.18162	0.036324	1.0108	0.4204
Residuals	54	1.94058	0.035937		
Color					
	Df	Sum	F	value	Pr(>F)
group	5	1853.4	370.67	9.5059	1.52 × 10${}^{-6}$
Residuals	54	2105.7	38.99		

**Table 4:** Multivariate normality test.

Normality Results				
Multivariate Normality				
	Test	Statistic	*p*-value	Result Normality
1	Mardia Skewness	418.922587	2.41 × 10${}^{-76}$	NO
2	Mardia Kurtosis	18.79618702	0	NO
Univariate Normality				
	Test	Statistic	*p*-value	Normality
1	Shapiro–Wilk	0.5681	<0.001	NO
2	Shapiro–Wilk	0.8662	<0.001	NO
3	Shapiro–Wilk	0.3137	<0.001	NO
4	Shapiro–Wilk	0.9175	6.00 × 10${}^{-4}$	NO

**Table 5:** Multivariate non-parametric test results.

Test	Test Statistic	df1	df2	*p*-Value
ANOVA type test *p*-value	7.805	17.961	193.98	0.001
Lawley00Hotelling test	11.937	20	104.977	0.001
Bartlett–Nanda–Pillai test	4.96	21.973	233.357	0.001
Wilks Lambda	7.858	20	170.098	0.001

## Data Availability

Upon request.

## Abbreviations

The following abbreviations are used in this manuscript:

PLA	Polylactic acid
MANOVA	Multivariate analysis of variance

## Appendix A

  Code

ssnonpartest(Tensile|Flexural|Weight|L$\tilde{T}$ime,ADT, test= c(1, 0, 0, 0),

alpha=0.05,factors.and.variables=TRUE) #ANOVA TYPE

Tensile|Flexural|Weight|$\tilde{T}$ime,ADT, test= c(0, 1, 0, 0),

alpha=0.05,factors.and.variables= TRUE) #Lawley Hotelling type)

ssnonpartest(Tensile|Flexural|Weight|L$\tilde{T}$ime,ADT, test= c(0, 0, 1, 0),

alpha=0.05,factors.and.variables = TRUE) # Bartlett–Nanda–Pillai type

ssnonpartest(Tensile|Flexural|Weight|L$\tilde{T}$ime,ADT, test= c(0, 0, 0, 1),

alpha=0.05,factors.and.variables = TRUE) # Wilks Lambda Type

## Appendix B

  Performing the Subsets of Algorithm based on the Factor levels

The hypothesis of equality between factor levels 0, 250, 500, 1000, 1500, 2000 is rejected

The hypothesis of equality between factor levels 250, 500, 1000, 1500, 2000 is rejected

The hypothesis of equality between factor levels 0, 500, 1000, 1500, 2000 is rejected

The hypothesis of equality between factor levels 0, 250, 1000, 1500, 2000 is rejected

The hypothesis of equality between factor levels 0, 250, 500, 1500, 2000 is rejected

The hypothesis of equality between factor levels 0, 250, 500, 1000, 2000 is rejected

The hypothesis of equality between factor levels 0, 250, 500, 1000, 1500 is rejected

The hypothesis of equality between factor levels 500, 1000, 1500, 2000 is rejected

The hypothesis of equality between factor levels 250, 1000, 1500, 2000 is rejected

The hypothesis of equality between factor levels 250, 500, 1500, 2000, is rejected

The hypothesis of equality between factor levels 250, 500, 1000, 2000 is rejected

The hypothesis of equality between factor levels 250, 500, 1000, 1500 is rejected

The hypothesis of equality between factor levels 0, 1000, 1500, 2000 is rejected

The hypothesis of equality between factor levels 0, 500, 1500, 2000 is rejected

The hypothesis of equality between factor levels 0, 500, 1000, 2000 is rejected

The hypothesis of equality between factor levels 0, 500, 1000, 1500 is rejected

The hypothesis of equality between factor levels 0, 250, 1500, 2000 is rejected

The hypothesis of equality between factor levels 0, 250, 1000, 2000 is rejected

The hypothesis of equality between factor levels 0, 250, 1000, 1500 is rejected

The hypothesis of equality between factor levels 0, 250, 500, 2000 is rejected

The hypothesis of equality between factor levels 0, 250, 500, 1500 is rejected

The hypothesis of equality between factor levels 0, 250, 500, 1000 is rejected

The hypothesis of equality between factor levels 1000, 1500, 2000 is rejected

The hypothesis of equality between factor levels 500, 1500, 2000 is rejected

The hypothesis of equality between factor levels 500, 1000, 2000 is rejected

The hypothesis of equality between factor levels 500, 1000, 1500 is rejected

The hypothesis of equality between factor levels 250, 1500, 2000 is rejected

The hypothesis of equality between factor levels 250, 1000, 2000 is rejected

The hypothesis of equality between factor levels 250, 1000, 1500 is rejected

The hypothesis of equality between factor levels 250, 500, 2000 is rejected

The hypothesis of equality between factor levels 250, 500, 1500 is rejected

The hypothesis of equality between factor levels 0, 1500, 2000 is rejected

The hypothesis of equality between factor levels 0, 1000, 2000 is rejected

The hypothesis of equality between factor levels 0, 1000, 1500 is rejected

The hypothesis of equality between factor levels 0, 500, 2000 is rejected

The hypothesis of equality between factor levels 0, 500, 1500 is rejected

The hypothesis of equality between factor levels 0, 500, 1000 is rejected

The hypothesis of equality between factor levels 0, 250, 2000 is rejected

The hypothesis of equality between factor levels 0, 250, 1500 is rejected

The hypothesis of equality between factor levels 0, 250, 1000 is rejected

The hypothesis of equality between factor levels 0, 250, 500 is rejected

The hypothesis of equality between factor levels 1500, 2000 is rejected

The hypothesis of equality between factor levels 1000, 2000 is rejected

The hypothesis of equality between factor levels 500, 2000 is rejected

The hypothesis of equality between factor levels 500, 1500 is rejected

The hypothesis of equality between factor levels 250, 2000 is rejected

The hypothesis of equality between factor levels 250, 1500 is rejected

The hypothesis of equality between factor levels 0, 2000 is rejected

The hypothesis of equality between factor levels 0, 1500 is rejected

The hypothesis of equality between factor levels 0, 1000 is rejected

The hypothesis of equality between factor levels 0, 500 is rejected

The hypothesis of equality between factor levels 0, 250 is rejected
